# Spatial prediction of malaria prevalence in Papua New Guinea: a comparison of Bayesian decision network and multivariate regression modelling approaches for improved accuracy in prevalence prediction

**DOI:** 10.1186/s12936-021-03804-0

**Published:** 2021-06-13

**Authors:** Eimear Cleary, Manuel W. Hetzel, Paul Siba, Colleen L. Lau, Archie C. A. Clements

**Affiliations:** 1 Research School of Population Health, Australian National University, Canberra, Australia; 2 Swiss Tropical and Public Health Institute, Basel, Switzerland; 3 University of Basel, Basel, Switzerland; 4 Papua New Guinea Institute of Medical Research, Goroka, Papua New Guinea; 5 Present Address: Centre for Health Research and Diagnostics, Divine Word University, Madang, Papua New Guinea; 6 School of Public Health, Faculty of Medicine, University of Queensland, Brisbane, Australia; 7 Faculty of Health Sciences, Curtin University, Bentley, Australia; 8 Telethon Kids Institute, Nedlands, Australia

## Abstract

**Background:**

Considerable progress towards controlling malaria has been made in Papua New Guinea through the national malaria control programme’s free distribution of long-lasting insecticidal nets, improved diagnosis with rapid diagnostic tests and improved access to artemisinin combination therapy. Predictive prevalence maps can help to inform targeted interventions and monitor changes in malaria epidemiology over time as control efforts continue. This study aims to compare the predictive performance of prevalence maps generated using Bayesian decision network (BDN) models and multilevel logistic regression models (a type of generalized linear model, GLM) in terms of malaria spatial risk prediction accuracy.

**Methods:**

Multilevel logistic regression models and BDN models were developed using 2010/2011 malaria prevalence survey data collected from 77 randomly selected villages to determine associations of *Plasmodium falciparum* and *Plasmodium vivax* prevalence with precipitation, temperature, elevation, slope (terrain aspect), enhanced vegetation index and distance to the coast. Predictive performance of multilevel logistic regression and BDN models were compared by cross-validation methods.

**Results:**

Prevalence of *P. falciparum,* based on results obtained from GLMs was significantly associated with precipitation during the 3 driest months of the year, June to August (β = 0.015; 95% CI = 0.01–0.03), whereas *P. vivax* infection was associated with elevation (β = − 0.26; 95% CI = − 0.38 to − 3.04), precipitation during the 3 driest months of the year (β = 0.01; 95% CI = − 0.01–0.02) and slope (β = 0.12; 95% CI = 0.05–0.19). Compared with GLM model performance, BDNs showed improved accuracy in prediction of the prevalence of *P. falciparum* (AUC = 0.49 versus 0.75, respectively) and *P. vivax* (AUC = 0.56 versus 0.74, respectively) on cross-validation.

**Conclusions:**

BDNs provide a more flexible modelling framework than GLMs and may have a better predictive performance when developing malaria prevalence maps due to the multiple interacting factors that drive malaria prevalence in different geographical areas. When developing malaria prevalence maps, BDNs may be particularly useful in predicting prevalence where spatial variation in climate and environmental drivers of malaria transmission exists, as is the case in Papua New Guinea.

## Background

Papua New Guinea (PNG), a Pacific island nation with a population of over 8 million people [1], has had a steady decline in malaria prevalence since 2004, when the national malaria control programme was awarded a Global Fund to Fight Aids, Malaria and Tuberculosis grant. This funding facilitated the free national distribution of long-lasting insecticidal nets (LLINs), improved diagnosis by rapid diagnostic tests (RDTs) and scaling up of artemisinin-based combination therapy (ACT) in all health facilities [2]. Consequently, *Plasmodium falciparum* and *Plasmodium vivax* prevalence in endemic areas (below 1600 m) has declined from 3.0 to 0.8%, and from 2.0 to 0.1% between 2010/11 and 2013/14, respectively [3]. In most parts of PNG, focus remains on controlling malaria, while a few areas such as the Highlands and selected islands may be amenable to sub-national elimination efforts [2, 4]. Despite this decline in prevalence, PNG still has the highest incidence of malaria in the Asia–Pacific Region, equal only in a global context to the highest-burden countries in sub-Saharan Africa [5]. As of 2016/2017, prevalence of *P. falciparum* in survey areas < 1600 m had rebounded to 4.8% and *P. vivax* to 2.6% [6].

The epidemiology of malaria varies considerably across the country and small-area spatial variation in malaria prevalence also exists [7, 8], attributed to a range of factors including varied uptake of interventions such as LLINs, as well as human behaviour and vector ecology [9, 10]. Environmental and climate factors associated with mosquito breeding sites and different vector dynamics, particularly between low-lying coastal areas and the highlands, also contribute to the variation in spatial patterns of malaria transmission [11, 12]. In the PNG lowlands, malaria transmission is perennial, with seasonal variation only in coastal areas where rainy and dry seasons are distinguishable [13]. In highland areas, marked seasonality exists where transmission is lower and unstable. In these areas, which are prone to seasonal epidemics or outbreaks and where populations lack acquired immunity, morbidity and mortality can be more severe [13]. The spatial distribution of both *P. falciparum* and *P. vivax* spans the entire country, however in terms of relative contribution to disease, *P. falciparum* is responsible for a greater proportion of infections in most settings [9]. There has been heterogeneity in the decline of *P. falciparum* and *P. vivax* prevalence in PNG, however, with a slower observed reduction in *P. vivax* due to transmission attributed to hypnozoite reservoirs of the *P. vivax* parasite [14].

Predictive prevalence maps based on spatial statistical models examining associations between environmental variables (often sourced using satellite remote sensing) and disease prevalence (often measured using surveys or surveillance data) are useful evidence-based decision tools for allocation of resources in control programmes [12, 15]. Spatial prevalence maps can reveal the geographical bounds of disease occurrence and variations in disease risk, including spatial changes in prevalence in response to control interventions [16–19]. By providing a better understanding of the epidemiology of disease over various spatial scales [20], they can help in the delivery of targeted control and elimination approaches, such as different combinations of interventions, adapted to the varying sub-national prevalence strata. These tools are of particular value in the context of constrained resources and in directing interventions to communities most in need of increased control efforts.

Predictive prevalence maps are often generated using the results of generalized linear models (GLM) that include environmental, demographic and intervention-related covariates. Such models can be developed at a range of spatial scales from global to local [21, 22]. However, challenges in using GLMs in the spatial prediction of malaria can be posed by spatial and temporal non-stationarity (where relationships between variables and correlation structures vary across a study area or time period), non-linear associations with covariates, spatial autocorrelation, and complex interactions between covariates, including collinearity [23]. All of these factors might limit the predictive accuracy of GLM-based approaches.

In recent years, graphical model-based approaches such as Bayesian decision networks (BDNs), have become more ubiquitous in infectious disease risk prediction, and used with good success [23, 24]. BDNs are graphical representations of variables, or nodes, in a system linked together to describe a network of interactions between explanatory variables and the outcome of interest [21, 25]. Variables are connected via directed arcs, indicating the direction of the association, with conditional probability tables quantify the relationship between each variable [25–28]. Such models can capture complex interactions of drivers of transmission and interacting nonlinear effects, and can provide quantitative representation of uncertainty in spatial predictions [21].

BDNs have been shown to have better prediction accuracy for malaria at high temporal and spatial resolutions compared with traditional methods (such as GLMs) [23], and have become increasingly popular for modelling of ecological and environmental systems [27, 29]. Both predictive accuracy and the ability to demonstrate uncertainty in predictions are beneficial for appropriately allocating resources when deciding where to implement control interventions. The objective of this paper was to produce national prevalence maps for *P. falciparum* and *P. vivax* infection prevalence in PNG, and to compare the predictive accuracy of GLM and BDN approaches for generating malaria prevalence maps in a complex environment.

## Methods

### Study setting

PNG is a Pacific Island nation consisting of the eastern half of New Guinea and a collection of several large and several hundred small islands [30]. At the last national census of population in 2011, the population of PNG was 7,275,324 people, 25.7% of which resided in the Momase Region, 15.1% in the Islands Region, 20.0% in the Southern Region and 39.2% in the Highlands Region. Considerable urban–rural and regional disparities exist in access to quality healthcare, and health infrastructure varies considerably between different regions. Poverty rates are high, with people of lower income being at a marked disadvantage in terms of health care access [31, 32]. Use of LLINs among people who have access to them is high (estimated to be approximately 78.7%) [33, 34], but access can be restricted due to poor infrastructure and remoteness of villages [35].

### Infection data

Data were collected as part of the national malaria indicator survey (MIS) conducted in 2010 and 2011 by the Papua New Guinea Institute of Medical Research. The survey was conducted in 77 randomly selected villages in 17 of the 20 provinces of PNG. In each village, 30 households were randomly selected for inclusion, to reflect village prevalence, and all present, consenting household members over 6 months of age were included as eligible for providing a blood sample for malaria diagnosis. Blood samples from 10,028 eligible participants were included in this analysis. Data collected included information on household use of LLINs, treatment-seeking behaviour relating to recent febrile illness, and parasite infection and species diagnosed by double-read light microscopy using capillary blood samples. Symptomatic survey participants were also tested by malaria RDT and, if positive, first-line antimalarial treatment was provided free of charge [3]. Village GPS coordinates and elevation above sea level were also recorded, and village-level point prevalence of *P. falciparum* and *P. vivax* were determined, using a Bernoulli distribution, based on light microscopy results. More detailed methods and results have been published elsewhere [3].

### Data on the physical environment

Average monthly precipitation and temperature data, aggregated over a 50-year period from 1950 to 2000, at 1 km^2^ resolution, were downloaded from the WorldClim website [36]. Elevation and slope (terrain aspect) data were extracted from a global digital elevation model (GDEM) obtained from the National Aeronautics and Space Administration (NASA) online repository of remote sensing image data, collected by the Advanced Spaceborne Thermal Emission and Reflection Radiometer (ASTER) aboard the Terra satellite [37]. Enhanced vegetation index (EVI) data were derived from the remote sensing images collected by the Moderate Resolution Imaging Spectroradiometer (MODIS), also aboard Terra. Distance to the coast was calculated using geographic information system (GIS) software by defining a coastline polygon and calculating the Euclidean distance from each cell on the map to the coast. All covariate data processing was carried out using ArcGIS software version 10.3 (ESRI, Redlands, California).

### Univariate analysis and variable selection

**Fig. 1:**
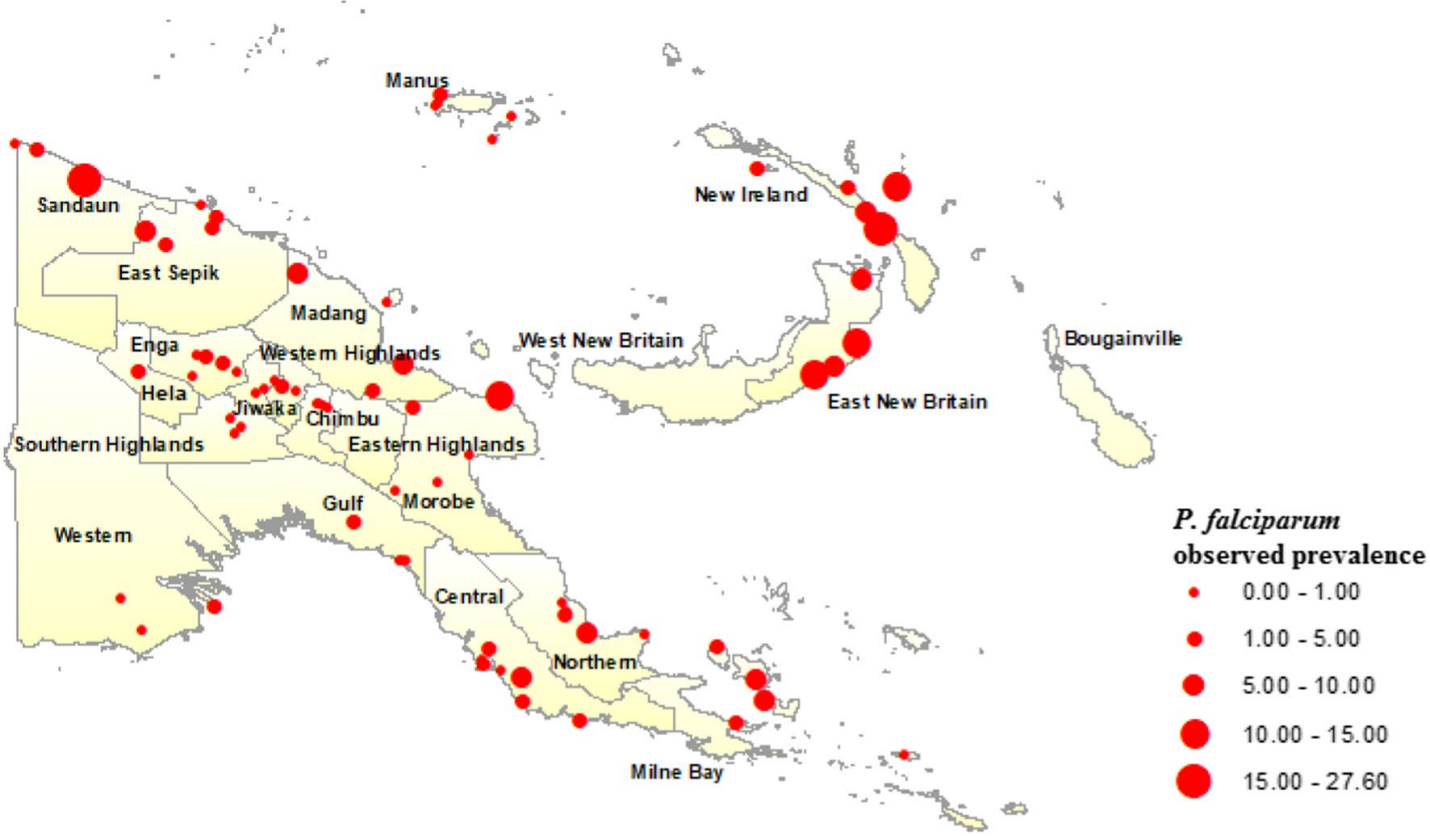
*Plasmodium falciparum* prevalence among 77 survey villages in Papua New Guinea, 2010/2011

**Fig. 2:**
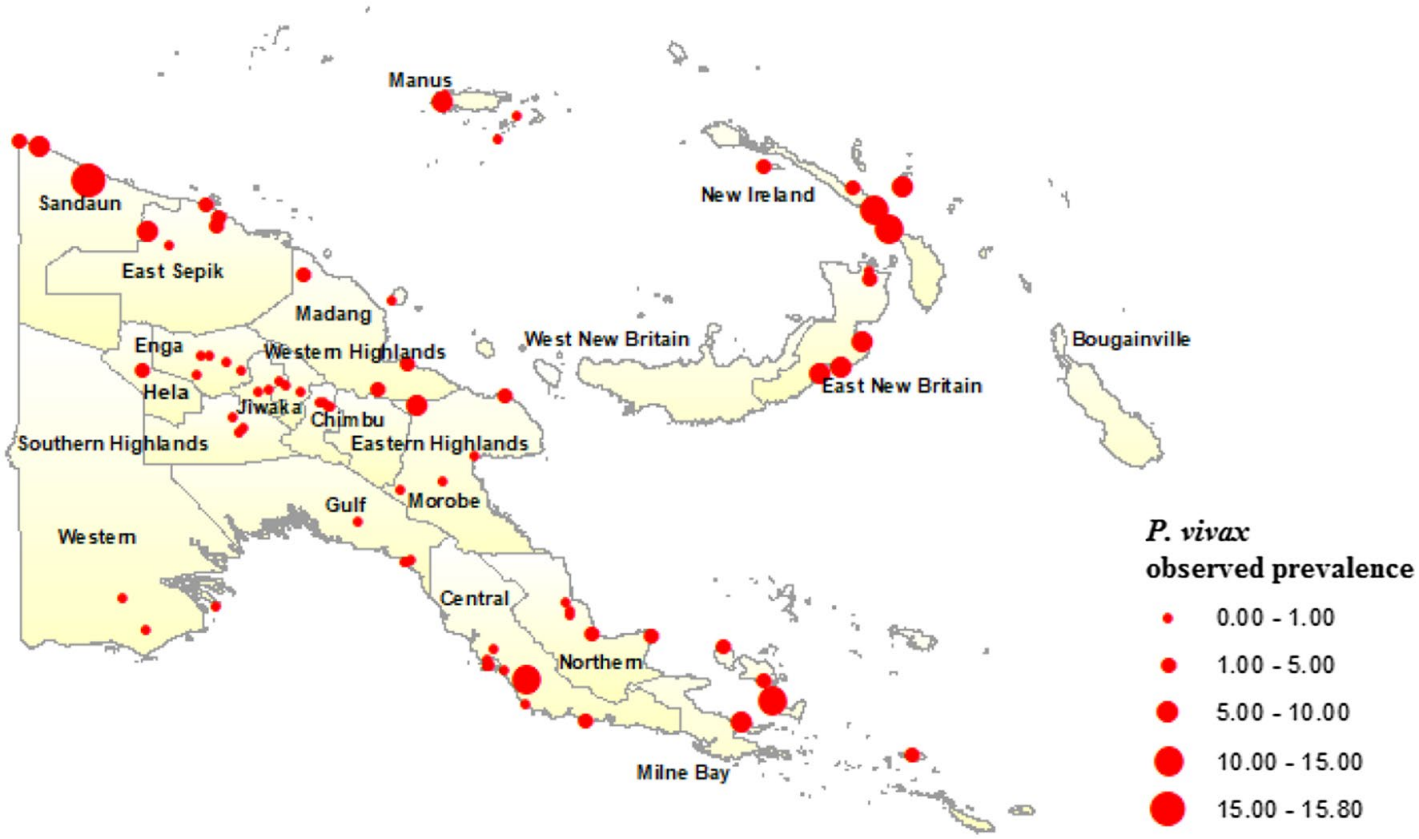
*Plasmodium vivax* prevalence among 77 survey villages in Papua New Guinea, 2010/2011

**Fig. 3:**
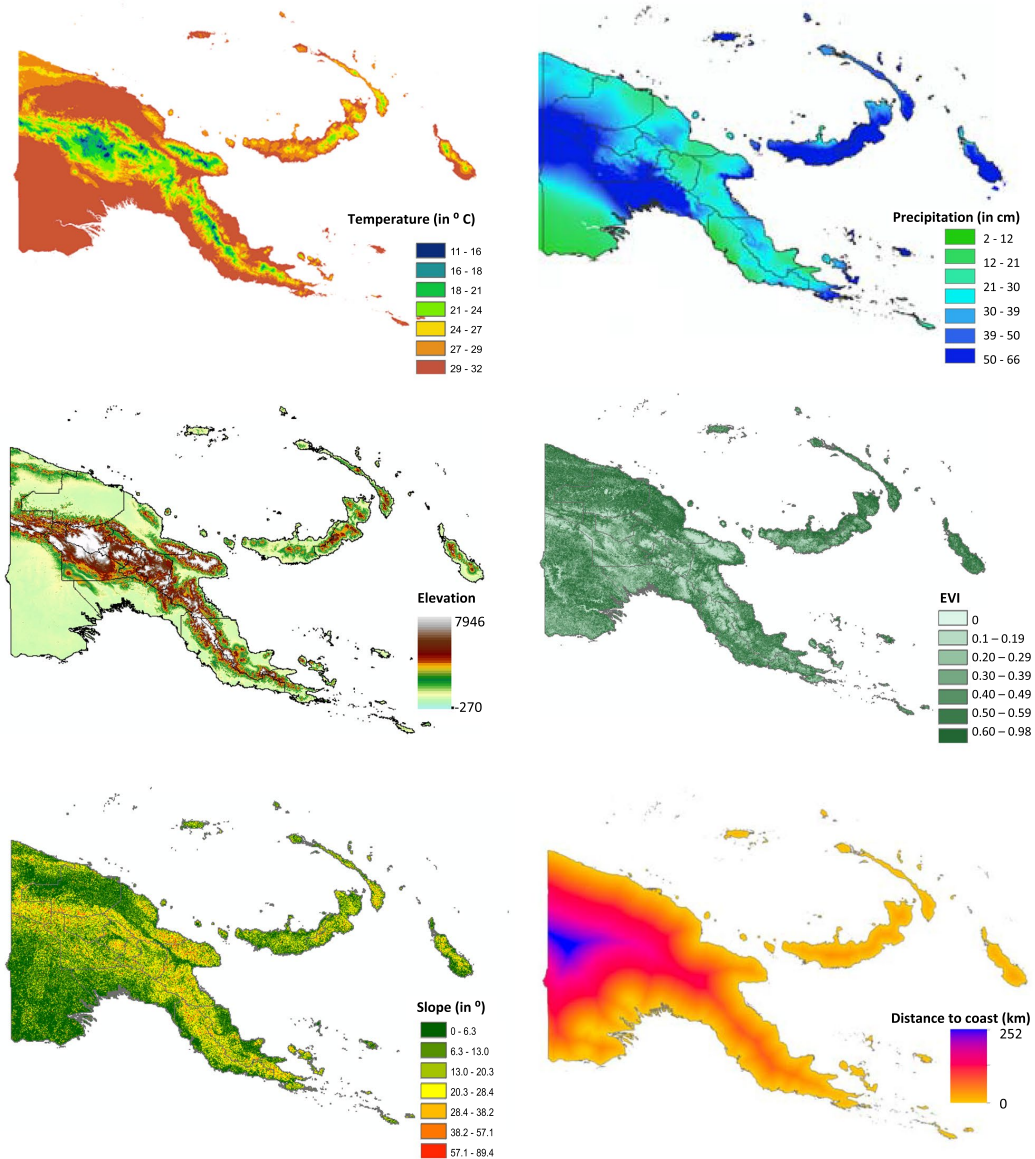
Spatial distribution of covariates associated with malaria transmission in Papua New Guinea. Average temperature and precipitation data were values for January aggregated over 50 years from 1950 to 2000

Maps of observed *P. falciparum* (Fig. 1) and *P. vivax* (Fig. 2) prevalence across the 77 surveyed villages in PNG were generated in ArcGIS and overlain with climate and environmental raster layers. Median values for temperature during the 3 hottest and coldest months (December to February, and June to August, respectively), precipitation during the wettest and driest 3 months (January to March, and June to August, respectively), EVI corresponding to the hottest and wettest (January) and coldest and driest (July) months of the year, slope (or terrain aspect), and elevation data were extracted to 5 km and 10 km buffer zones around the centre point of each survey village location (Fig. 3). The Euclidean distance from each village centroid point to the coastline of PNG was also calculated and values extracted. All data management and extraction was carried out using ArcGIS software.

Bivariate associations of *P. falciparum* and *P. vivax* prevalence with environmental and climate data extracted to both 5 km and 10 km buffer zone were determined by logistic regression analyses that accounted for clustering at the village level. Variables were selected for inclusion in further analyses based on a *p*-value of < 0.05 and lowest value of the Akaike Information Criterion (AIC). Collinearity of explanatory variables was assessed using a tolerance cut point of < 0.02 and variance inflation factor (VIF) cut-off value of > 5. The only variables found to exhibit collinearity were temperature and elevation and the most appropriate variables for inclusion in multivariate regression models were selected on the basis of the lowest AIC value. All bivariate and multivariate regression analyses were carried out using Stata statistical software version 14 (StataCorp, College Station, Texas).

### Multivariable generalized linear models

Multilevel mixed-effects logistic regression models were developed for *P. falciparum* and *P. vivax* using selected variables. Proportion of bed net use in each village (survey data included information on ownership of any type of net and of LLIN), age, gender, wealth quintile (the calculation of which has been described elsewhere [33]) and annual quarter during which the survey was carried out were included in the models to adjust for confounding of associations with environmental variables. Separate multivariable models were built for each *Plasmodium* species and final models were selected based on the lowest AIC value. Semivariograms of the regression model residuals were plotted using the R open source software version 3.2.2 (R Foundation for Statistical Computing, Vienna, Austria) in order to identify spatial autocorrelation. As there was no evidence of spatial autocorrelation from the semivariograms fitted to the residuals of a multivariable fixed-effects model, we were unable to fit spatial GLMs using model-based geostatistics. Spatially-explicit GLMs were therefore not developed further. Multivariable models for each parasite species were also developed specific to each of the four geographic regions of PNG to examine the complexity of drivers of malaria transmission in PNG. Due to small sample numbers retained by disaggregating the national survey dataset by region, the results of these models are not presented here. The different drivers of malaria transmission observed between these models, however, informed the inclusion of the regional variable in BDN models to account for this complexity.

### Spatial prevalence prediction and generation of prevalence maps using generalized linear modelling results

Spatial prevalence predictions were made, using environmental and climate fixed effects only, by multiplying values for each cell of the environmental variable raster layers in the model by the corresponding covariate coefficient from the multilevel regression models, adjusted for confounding variables described above. The resulting raster layer values were summed (together with the intercept) and the logit back-transformation calculated using the map algebra tool in ArcGIS. Although not all variables in *P. falciparum* and *P. vivax* multilevel mixed-effects models were found to be significant upon regression analysis, all were retained for generation of predicted prevalence maps so as not to exclude any potential explanatory data.

The equations for spatial prevalence prediction (*p*) in each location (*i*) of *P. falciparum* and *P. vivax* are as follows:

$$\begin{aligned} P.{\text{ }}falciparum:{\text{logit }}\left( {{\text{p}}_{{\text{i}}} } \right) & = - 59.467 - 0.01*{\text{Enhanced}}\;{\text{vegetation}}\;{\text{index}}\;{\text{in}}\;{\text{July}}_{{\text{i}}} \\ & \quad {-}{\text{ }}0.073*{\text{Distance}}\;{\text{to}}\;{\text{coast}}_{{\text{i}}} + 4.77*{\text{Maximum}}\;{\text{temperature }}\left( {{\text{December}}\;{\text{to}}\;{\text{February}}} \right)_{{\text{i}}} \\ & \quad {-}{\text{ }}0.09*{\text{Maximum}}\;{\text{temperature}}\left( {{\text{December}}\;{\text{to}}\;{\text{February}}} \right)_{{\text{i}}} ^{2} + 0.01*{\text{Precipitation }}\left( {{\text{June}}\;{\text{to}}\;{\text{August}}} \right)_{{\text{i}}} \\ \end{aligned}$$

$$\begin{aligned} P.{\text{ }}vivax:{\text{logit}}\left( {{\text{p}}_{{\text{i}}} } \right) & = - 5.941 + 0.04*{\text{Enhanced}}\;{\text{vegetation}}\;{\text{index}}\left( {{\text{January}}} \right)_{{\text{i}}} {-}1.33*{\text{Distance}}\;{\text{to}}\;{\text{coast}}_{{\text{i}}} \\ & \quad {-}0.26*{\text{Elevation}}_{{\text{i}}} + 0.01*{\text{Precipitation}}\left( {{\text{June}}\;{\text{to}}\;{\text{August}}} \right)_{{\text{i}}} + 0.12*{\text{Slope}}_{{\text{i}}} \\ \end{aligned}$$

### Bayesian decision network models

A BDN model structured based on the biological assumptions underlying malaria transmission in PNG, and associations with ecological covariates obtained upon initial univariate analysis, were compiled using Netica software version 5.24 (Norsys Software Corp., Vancouver, Canada) and the bnlearn package [38] in R statistical software. The variables found to have strongest associations with the outcome, based on AIC criterion, were placed closest to the parent node and sensitivity to findings analyses were conducted in Netica software to verify appropriate positioning of variables in the network. Sensitivity to findings analysis verifies which nodes in the model are most informative in making predictions for the outcome of interest. Quantile, Hartemink and equal interval methods were explored for discretization of continuous predictor variables in the model in R software. The equal interval method of discretization was found to be the optimal discretization methodology for the models used here.

Conditional probability tables (CPTs) were generated to quantify the relationships between explanatory variables and the outcome variable. CPTs and predicted probability of the outcome [39] were based on data entered into the model and a priori beliefs were updated through belief propagation using Bayes’ Theorem (posterior = likelihood * prior/probability of evidence) [25, 28]. A priori beliefs relate to the logical structure of explanatory nodes in the BDN model and a priori probabilities are updated as new knowledge about the systems is obtained (observational data on which the model is learned and CPTs are produced) making them posterior beliefs [40].

### Spatial prevalence prediction and generation of prevalence maps using Bayesian decision network model results

Predicted *P. falciparum* and *P. vivax* prevalence were determined according to CPTs from the BDN model and predictions were made for each spatial point on a continuous gridded vector layer of environmental and climate measures for PNG using the bnspatial package in R open source statistical software. The spatial distributions of these predicted probabilities were plotted in ArcGIS and the resulting gridded point maps smoothed using the inverse weighting function. Maps showing degree of spatial entropy representing uncertainty for both models were also produced in the bnspatial package.

### Model validation

The predictive accuracy of GLM and BDN *P. falciparum* and *P. vivax* models was assessed using Receiver operating characteristic (ROC) curves and Area under the curve (AUC) values obtained using predicted prevalence (*p*_i_) values against observed adjusted village level prevalence values. For GLM validation, observed prevalence values were adjusted for age, gender, wealth quintile, bed net use and season during which the surveys were calculated using the dstdize command in Stata statistical software. Cross validation was carried out by defining four randomly selected training and test dataset subsets. Training data subsets represented 75% of the full dataset, with the remaining 25% of the full dataset retained as test data subsets. Models were run on training datasets, and predictions made for the remaining 25% test dataset. Observed and predicted values made for each randomly selected training and test dataset were then combined, and model prediction accuracy was determined by generating ROC curves and AUC values of predicted values versus observed prevalence values. The spatial pattern of entropy, or uncertainty in prevalence prediction, was examined by generating maps of Shannon index values using the bnspatial package in R open source statistical software.

## Results

### Demographics

The 10,028 survey participants from 77 randomly selected villages included a slightly higher proportion of female than male participants (52.74%), with the largest proportion of participants in the > 18 year-old age category (51.26%) compared with 17.69% of participants in the ≤ 5 year-old age category. Below an altitude of 1600 m, where historically malaria has been endemic in PNG [3, 13], *P. falciparum* prevalence, as determined by microscopy, (2.75%; 95% CI 2.45–3.09%) was slightly higher than *P. vivax* prevalence (2.05%; 95% CI 1.79–2.35%; Table1). The highest observed village-level prevalence of *P. falciparum* and *P. vivax* were 27.6% and 15.8%, respectively.

**Table 1.** 2010/2011 national malaria control intervention and prevalence of parasitaemia household survey results

Variable	N (%)
*P. falciparum* < 1600 m	276 (2.75)
*P. vivax* < 1600 m	206 (2.05)
Gender	
Female	5271 (52.74)
Male	4723 (47.26)
Age (years)	
0–5	1772 (17.69)
6–18	3111 (31.05)
19–99	5136 (51.26)
Bed net use	
No	4660 (46.47)
Yes	5368 (53.53)

*Plasmodium falciparum* and *Plasmodium vivax* prevalence determined by microscopy among 10,028 individuals surveyed in the 2010/2011 national malaria control intervention and prevalence of parasitaemia household survey

Geographically, the highest prevalence of both *P. falciparum* (Fig. 1) and *P. vivax* (Fig. 2) were observed in the islands of East New Britain and New Ireland, the north coast and on the Papuan Peninsula in the east of the country. Prevalence of *P. falciparum* and *P. vivax* was not ascertained for the Eastern Highlands, West New Britain and the Autonomous Region of Bougainville, however, as the 2010/2011 survey did not include data points for these areas. Overall, 53.53% of participants reported bed net use, which included LLIN and use of any net type, with village-level net use ranging from 8.47 to 93.7%. Further details pertaining to the demographics associated with infections within both malaria species are described elsewhere [3].

### Generalized linear models

Among the environmental predictor variables, *P. falciparum* was found only to be significantly associated with precipitation during the 3 driest months of the year, June to August (*β* = 0.015; 95% CI = 0.01–0.03; Table 2), whereas *P. vivax* infection at village level was associated with elevation (*β* = − 0.26; 95% CI = − 0.38 to − 3.04), precipitation during the 3 driest months of the year (*β* = 0.01; 95% CI = 0.01–0.02) and slope (*β* = 0.12; 95% CI = 0.05–0.19). In terms of demographics, highest wealth quintile was negatively associated with *P. falciparum* prevalence (*β* = − 0.89; 95% CI = − 1.62 to − 0.016), as was age, with participants aged between 5 and 18 years (*β* = − 0.33; 95% CI = − 0.53 to − 0.12), and adults over 18 years of age (*β* = − 1.27; 95% CI = − 1.62 to − 0.92) at lower prevalence compared with children under five. *P. falciparum* prevalence was also associated with season during which the survey was conducted, with a higher prevalence between March and May (*β* = 1.11; 95% CI = 0.33–1.88) compared with November to February. *P. vivax* was associated with age, with lowest prevalence among the participants over 18 years of age (*β* = − 1.55; 95% CI = − 2.08 to − 1.02).

**Table 2:** Results of generalized linear multivariable regression models exploring associations of *Plasmodium falciparum* and *Plasmodium vivax* prevalence with climate and environmental covariates

*P. falciparum*		*P. vivax*	
Variable	Β (95% CI)	Variable	Β (95% CI)
Enhanced Vegetation Index Jan	− 0.01 (− 0.07 to 0.06)	Enhanced vegetation Index Jan	0.04 (− 0.02 to 0.09)
Distance to the coast	− 0.73 (− 2.73 to 0.77)	Distance to the coast	1.33 (− 0.01 to 2.98)
Tmax Dec to Feb (hottest)	4.77 (− 1.10 to 9.97)	**Elevation**	− **0.26 (**− **0.38 to **− **3.04)**
Tmax sq	− 0.09 (− 0.19 to 0.02)	**Precipitation Jun to Aug (driest)**	**0.01 (0.01 to 0.02)**
**Precipitation Jun to Aug (driest)**	**0.01 (0.01 to 0.03)**	**Slope**	**0.12 (0.05 to 0.19)**
Proportion of bednet use per village	0.91 (− 0.23 to 2.11)	Proportion of bednet use per village	− 0.45 (− 1.78 to 0.88)
Female	− 0.14 (− 0.37 to 0.09)	Female	− 0.04 (− 0.40 to 0.31)
Wealth quintile 2	0.30 (− 0.15 to 0.75)	Wealth quintile 2	− 0.06 (− 0.39 to 0.50)
Wealth quintile 3	− 0.06 (− 0.57 to 0.45)	Wealth quintile 3	0.16 (− 0.41 to 0.74)
Wealth quintile 4	− 0.36 (− 0.83 to 0.09)	Wealth quintile 4	− 0.24 (− 0.81 to 0.33)
**Wealth quintile 5**	− **0.89 (**− **1.62 to **− **0.16**)	Wealth quintile 5	− 0.79 (− 1.57 to − 0.15)
**Age > 5–18**	− **0.33 (**− **0.53 to **− **0.12)**	**Age > 5–18**	− **0.67 (**− **1.10 to **− **0.25)**
**Age > 18+**	− **1.27 (**− **1.62 to **− **0.92)**	**Age > 18+**	− **1.55 (**− **2.08 to **− **1.02)**
**Season Mar–May**	**1.11 (0.33 to 1.88)**		
Season Jun–Aug	0.74 (− 0.11 to 1.59)		

Covariates highlighted in bold are representative of covariates found to be significantly associated with the outcome variable upon generalised linear modelling

### Bayesian decision network models

**Fig. 4:**
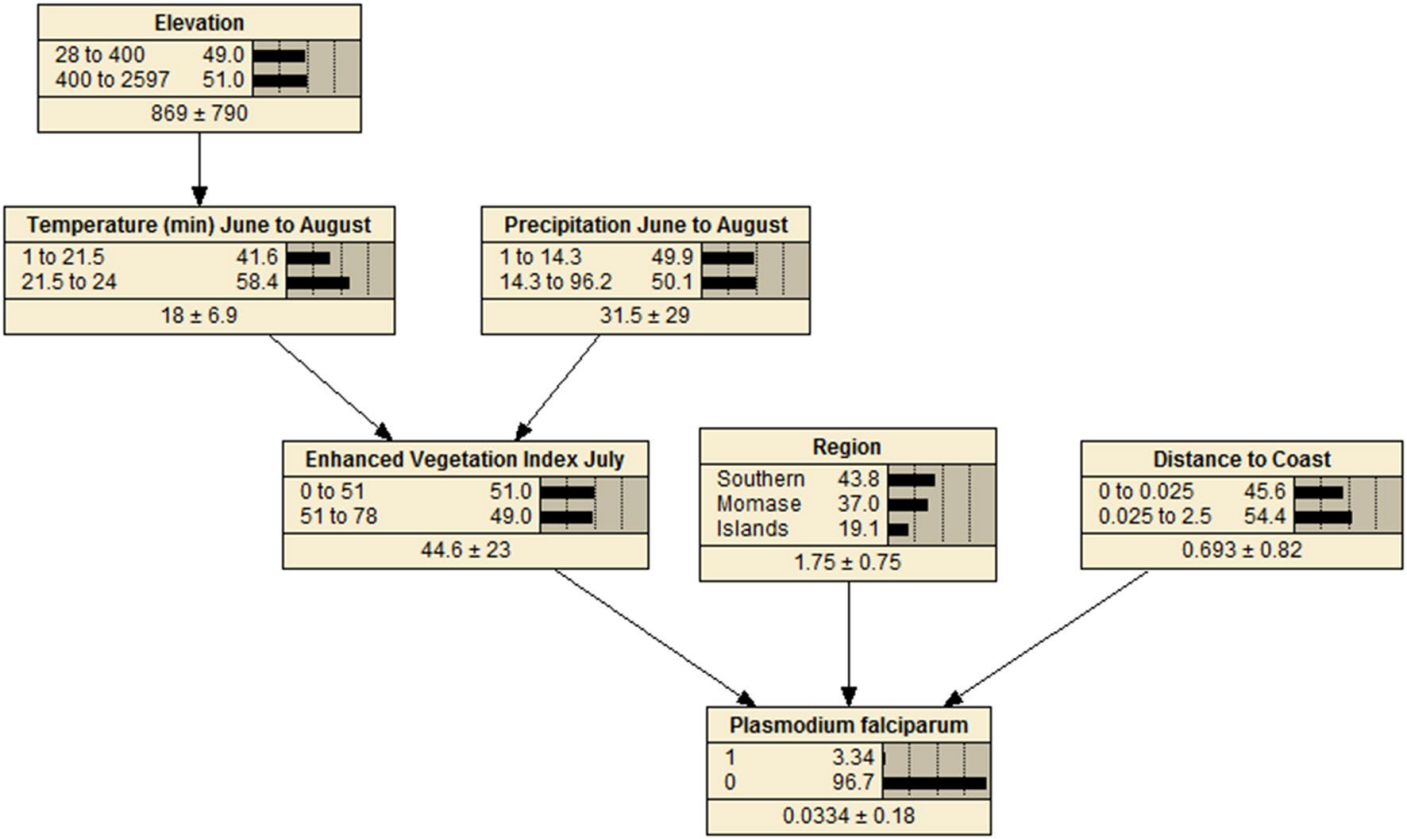
Bayesian decision network showing associations of environmental variables with *Plasmodium falciparum* prevalence in PNG

**Fig. 5:**
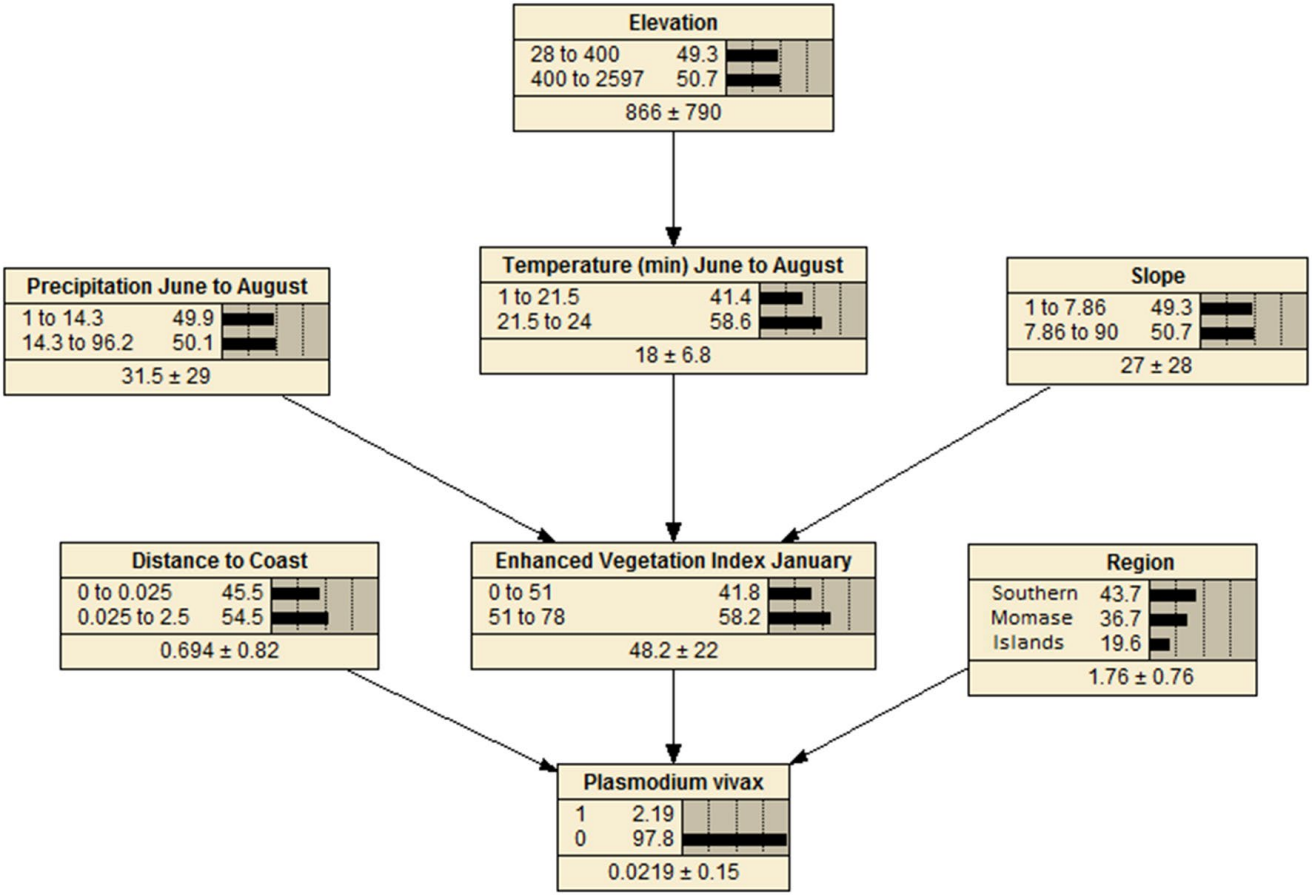
Bayesian decision network showing associations of environmental variables with *Plasmodium vivax* prevalence in PNG

**Fig. 6:**
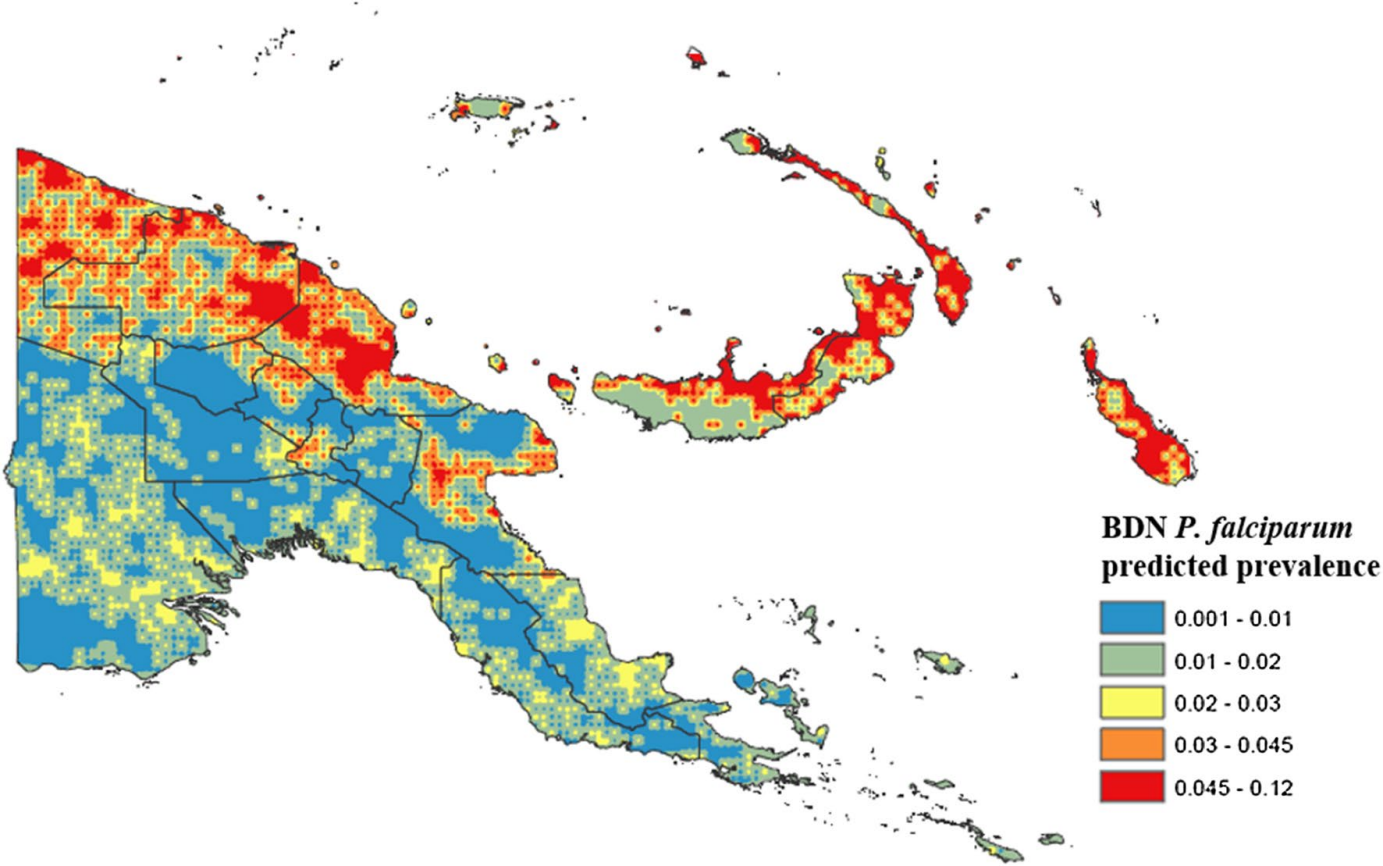
Predicted spatial distribution of *Plasmodium falciparum* in Papua New Guinea based on BDN model results

BDNs for both *P. falciparum* (Fig. 4) and *P. vivax* (Fig. 5) were structured with EVI, region and distance to the coastline variables positioned with arcs directly related to disease prevalence, as these variables were found to be the strongest predictors of both *P. falciparum* and *P. vivax* prevalence. Spatial prevalence maps showing the predicted distribution of *P. falciparum* (Fig. 6) based on the results of the BDN models predict the prevalence of *P. falciparum* prevalence to be highest in the Islands Region provinces of PNG, New Ireland and New Britain (0.03 to 0.12), consistent with the results of the observed prevalence in the national malaria household survey. Consistent with observed prevalence, a higher predicted *P. falciparum* prevalence was also seen along the northern coast in the provinces of Sandaun, East Sepik and Madang (0.03 to 0.12), relative to the Highlands Region and south coast of PNG.

**Fig. 7:**
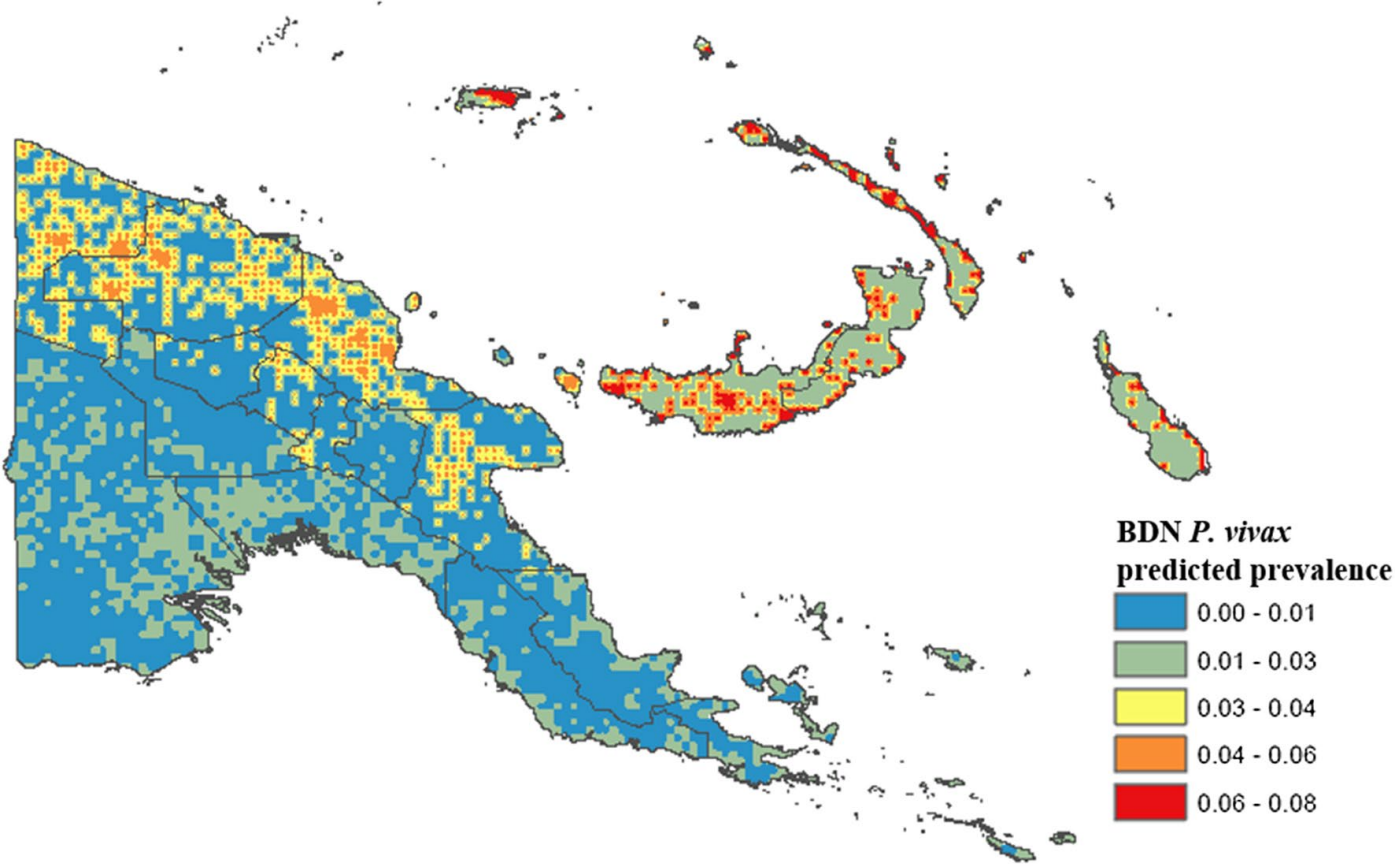
Predicted spatial distribution of *Plasmodium vivax* in Papua New Guinea based on BDN model results

Average *P. falciparum* predicted prevalence was lower in the Highlands Region provinces (0.001 to 0.03), along the south coast (0.001 to 0.03), where population density is sparser, and in Milne bay (0.001 to 0.03). The predicted prevalence of *P. vivax* (Fig. 7) was also highest in the islands, ranging from 0.01 to 0.08. The highest predicted prevalence of *P. vivax* prevalence was observed along the north coast (0.03 to 0.08) consistent with highest observed prevalence (3.0% and 6.0%). Predicted *P. vivax* prevalence was lowest along the south coast (0.00 to 0.03) and in the Highlands Region (0.00 to 0.03), similar to patterns observed for *P. falciparum*.

### Model validation

**Fig. 8:**
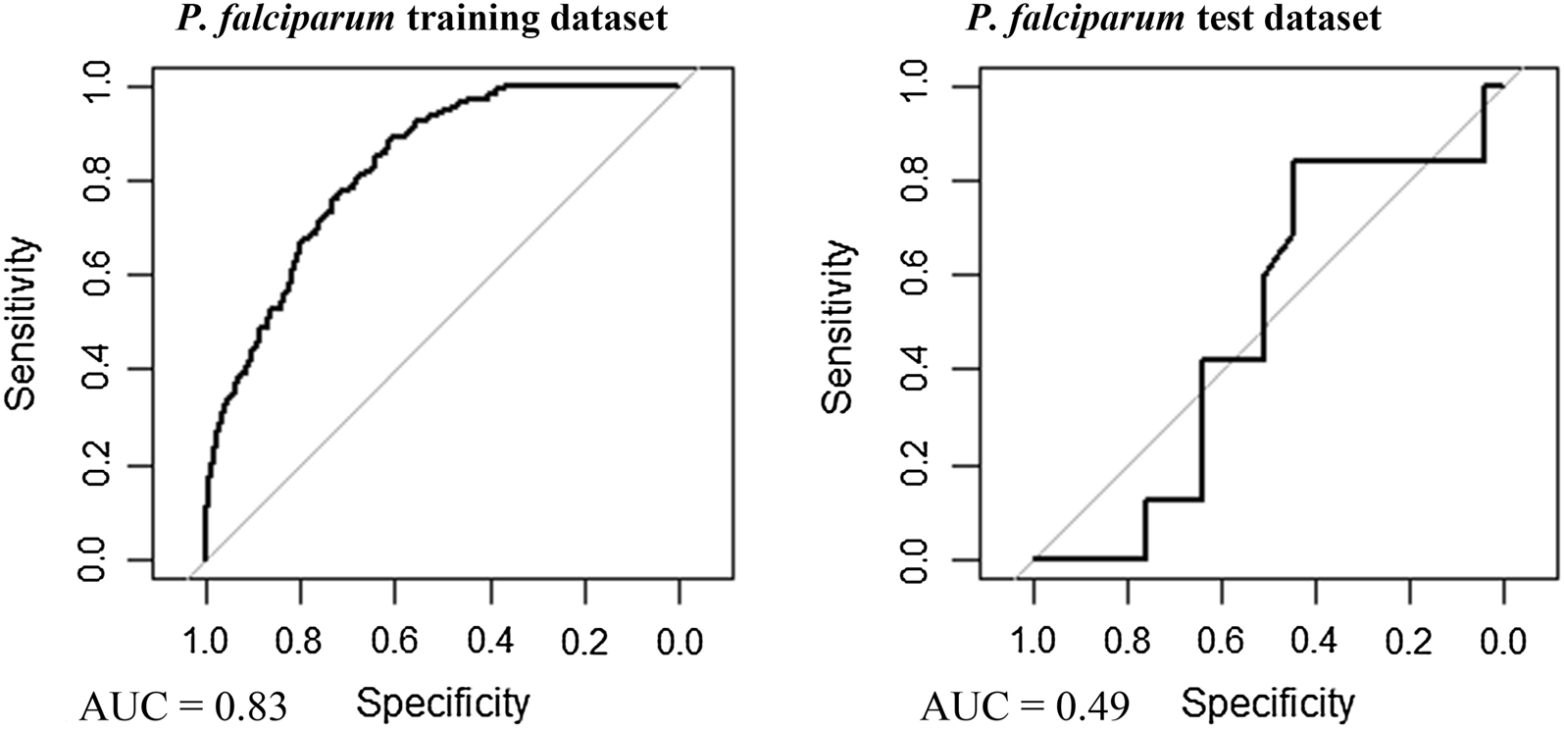
Receiver operating curves showing predictive performance of *Plasmodium falciparum* GLMs

**Fig. 9:**
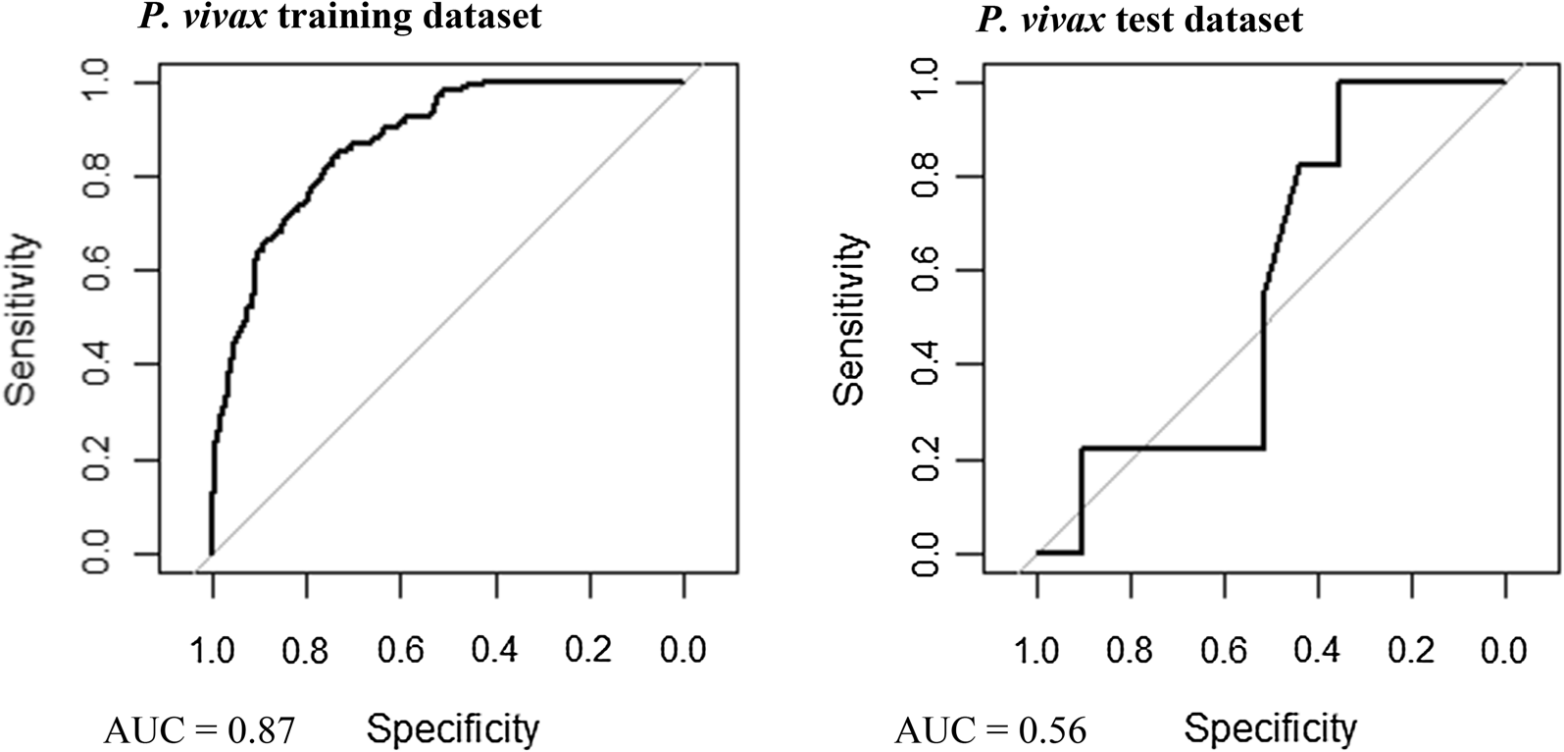
Receiver operating curves showing predictive performance of *Plasmodium vivax* GLMs

**Fig. 10:**
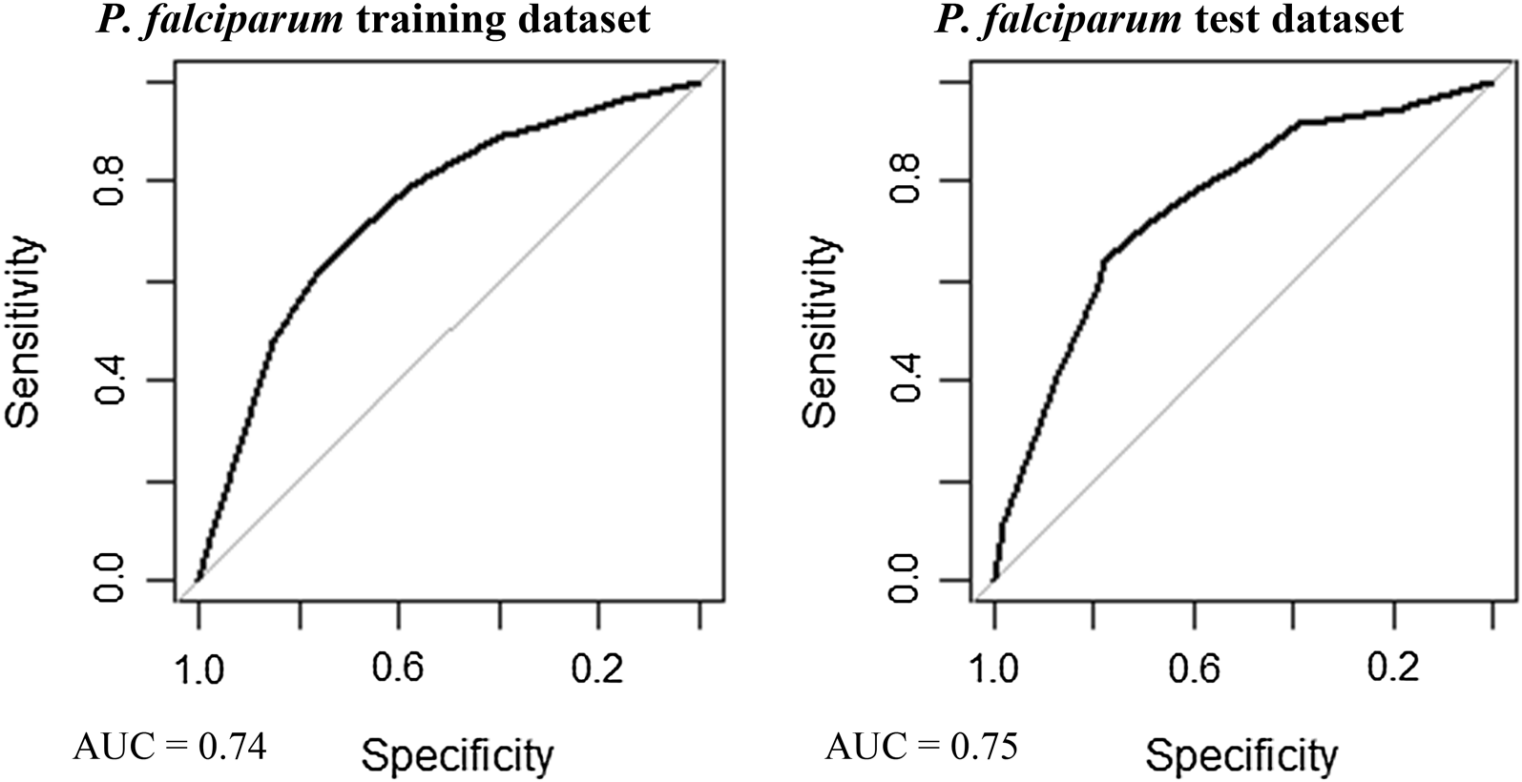
Receiver operating curves showing predictive performance of *Plasmodium falciparum* BDNs

**Fig. 11:**
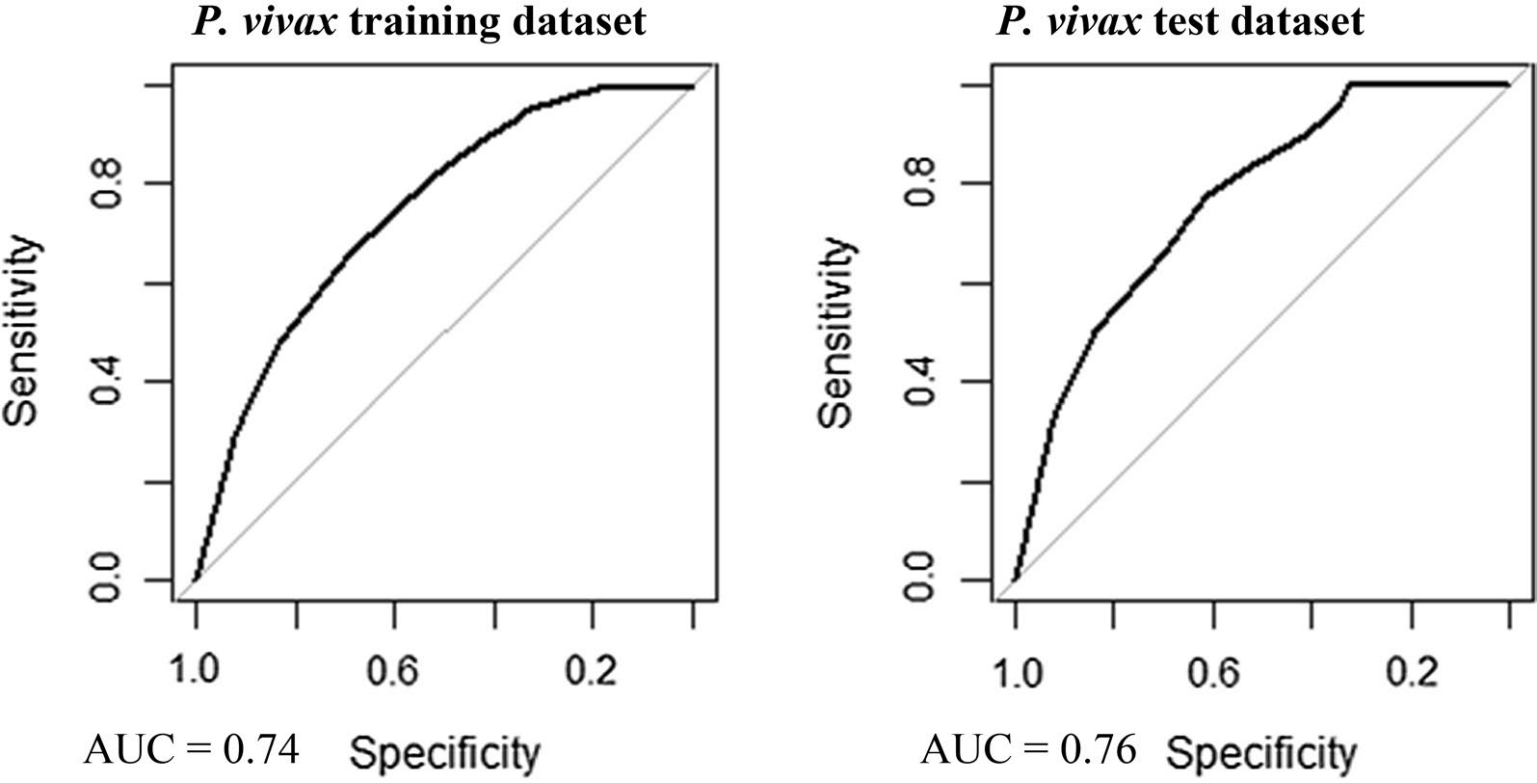
Receiver operating curves showing predictive performance of *Plasmodium vivax* BDNs

**Fig. 12:**
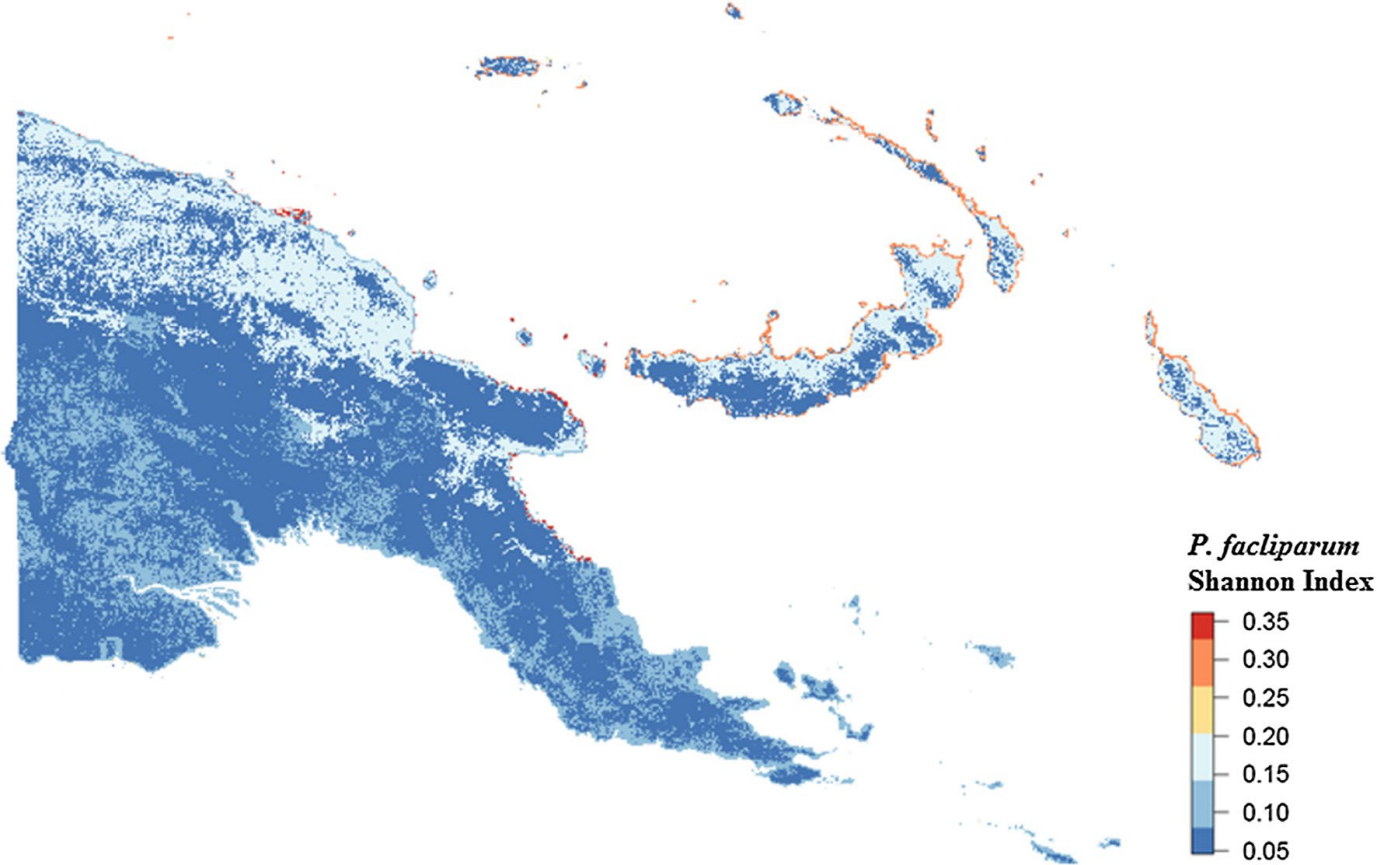
Spatial distribution of Shannon Index measure of entropy or uncertainty for predicted prevalence of *Plasmodium falciparum* using Bayesian decision network model

**Fig. 13:**
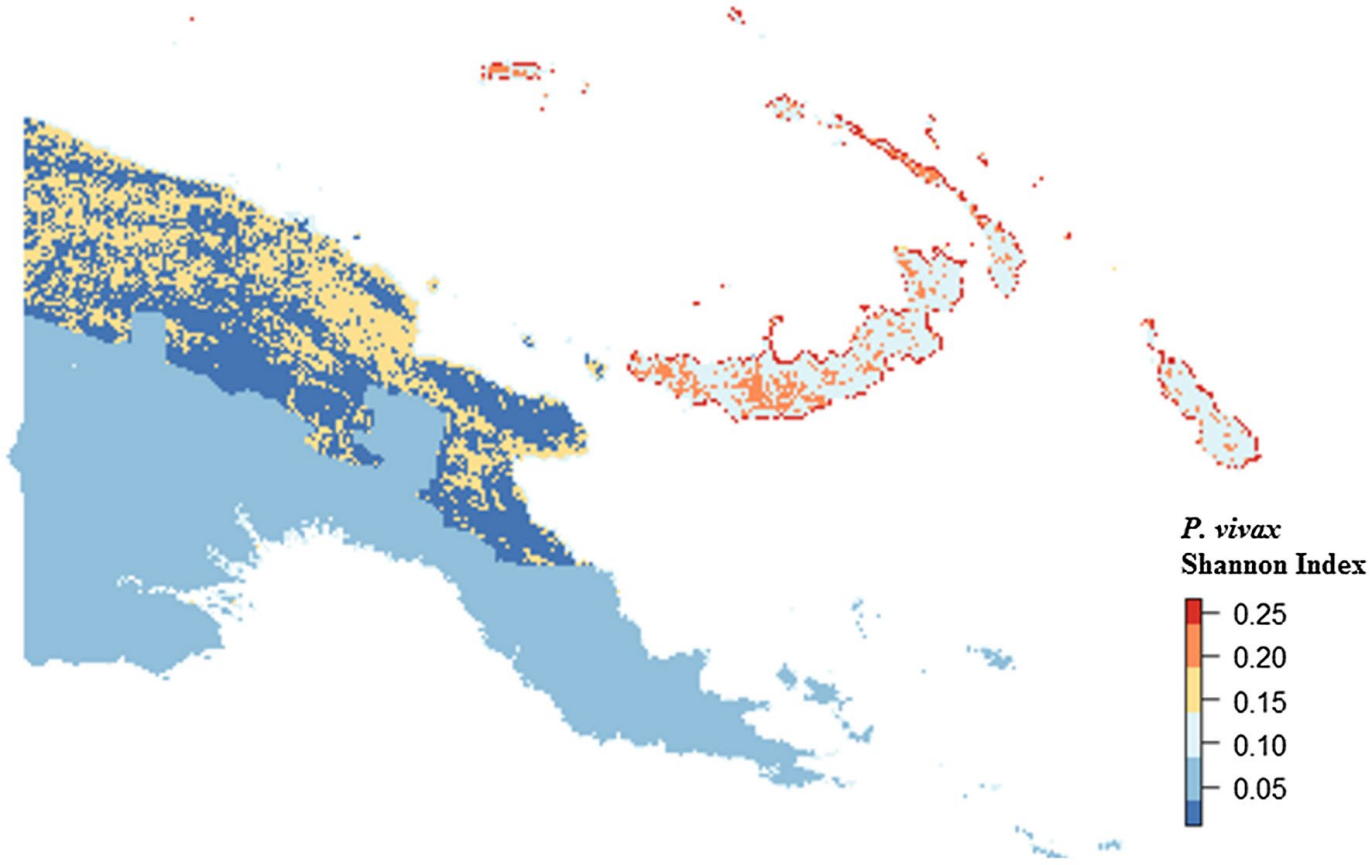
Spatial distribution of Shannon Index measure of entropy or uncertainty for predicted prevalence of *Plasmodium vivax* using Bayesian decision network model

Multivariable, multilevel GLMs were found to have good predictive performance using models run on training datasets (*P. falciparum* AUC = 0.83, *P. vivax* AUC = 0.87; Table 3, Figs. 8 and 9). Predictions made on test datasets however exhibited unsatisfactory agreement for *P. falciparum* and *P. vivax*, with the predicted prevalence not performing much better than random allocation of status (*P. falciparum* AUC = 0.49, *P. vivax* AUC = 0.56). Predictions made from BDN models run using training datasets exhibited slightly poorer prediction accuracy compared with GLMS (*P. falciparum* AUC = 0.74, *P. vivax* AUC = 0.74; Figs. 10 and 11)*.* Predictions made on test datasets using BDN models run on training datasets, however, were found to have improved accuracy compared with GLMs (*P. falciparum* AUC = 0.75, *P. vivax* AUC = 0.76) and good agreement between predictions made on training and test datasets. The spatial pattern of entropy, determined by Shannon index values, had a similar distribution to the spatial distribution of highest predicted prevalence of *P. falciparum* (Fig. 12) and *P. vivax* (Fig. 13), reflecting higher standard errors for higher predicted prevalence.

**Table 3:** Area under the curve values for GLM and BDN models

	AUC values			
	*P. falciparum*		*P. vivax*	
GLM village level cross-validation AUC values	Training dataset	Test dataset	Training dataset	Test dataset
	0.83	0.49	0.87	0.56
BDN village level cross-validation AUC values	Training dataset	Test dataset	Training dataset	Test dataset
	0.74	0.75	0.74	0.76

Area under the curve (AUC) results of Receiver Operating Characteristic (ROC) cross validation for generalised linear regression (GLM) models and Bayesian decision network (BDN) models

## Discussion

This study showed that BDN models provided improved accuracy in the spatial prediction of malaria in PNG compared with the more commonly used GLM approach. The reasons for this improvement in predictive accuracy may lie in the ability of BDN models to retain collinear variables thus utilizing more information for model predictions. Also, the ability of BDNs to incorporate complex interactions between explanatory variables in the model enables links between all variables (both explanatory and outcome), rather than only defining associations between each explanatory variable and the outcome independently [41]. The findings presented here are consistent with other studies which have shown improved accuracy in the prediction of malaria parasitaemia using BDNs [42], in modelling the complex interactions of leptospirosis transmission in Fiji [24] and in assessing the prevalence of Murray Valley encephalitis in Western Australia [43].

The predicted spatial distribution of *P. falciparum* and *P. vivax* were found to correspond with observed prevalence determined from a national survey of malaria prevalence in PNG and with environmental covariates well established as drivers of malaria transmission. *Plasmodium falciparum* predicted prevalence was found to be highest along the northern coast of PNG in Sandaun, East Sepik and Madang provinces, which is consistent with higher average annual temperatures and precipitation observed in these provinces. A lower *P. falciparum* predicted prevalence observed in Morobe and along the southern coast also corresponded to lower average annual temperatures and rainfall, as well as to lower population density [44]. The Western Highlands province, Chimbu, Enga, and the Islands provinces of New Britain and New Ireland had a higher predicted *P. falciparum* predominance, which is consistent with a higher observed prevalence determined from survey results (Fig. 1). Predicted prevalence in West New Britain was found to be slightly lower than in East New Britain, where elevation and vegetation index values are lower.

The predicted spatial distribution of *P. vivax* was found to have a similar distribution pattern to *P. falciparum* predicted prevalence. A higher *P. vivax* predicted prevalence was observed along the north coast and in the Islands provinces of East and West New Britain, New Ireland and Manus Island. These results are consistent with survey results which found higher prevalence of *P. vivax* malaria on the northern PNG coast, as well as in the outer islands. The higher predicted prevalence in these provinces also corresponds to higher average temperature, EVI and population density, and with lower elevation. A lower predicted *P. vivax* prevalence was observed along the southern coastline, and in the highlands, following a similar distribution to *P. falciparum* malaria and to observed prevalence determined by survey results. *Plasmodium vivax* prevalence was also predicted to be lower in Milne Bay and Central province, however, which is contradictory to survey findings.

Drivers of malaria transmission across PNG vary spatially and, therefore, a single, stationary model of environmental and climate predictors (such as the multilevel GLM presented here) was not found to be appropriate for spatial prediction of malaria prevalence. For example, while temperature may be a significant driver of transmission in the lowlands or coastal areas, altitude may be a better predictor of malaria prevalence in the highlands. Using BDN models therefore allows for improved accuracy in the spatial predictions of malaria prevalence to be determined where non-stationarity in drivers of transmission exists. This lies in the ability of these models to generate predictions based on complex interactions of environmental covariates associated with infectious disease transmission. An additional benefit of BDNs is that they can be used to predict malaria prevalence under defined scenarios and specific parameters of explanatory variables in the model, for example under highest range of temperature and precipitation values. This may be useful for predicting the spatial distribution of malaria under future climate and environmental scenarios, or for incorporating variables to predict disease distribution associated with the effects of control interventions.

The visual nature of BDNs in presenting graphical interactions of environmental covariates associated with infectious disease transmission lend themselves to being easily interpretable in population health communication and in demonstrating different explanations of the outcome [28, 43]. In Vietnam, for example, BDNs have been used for communication of mitigation and public health strategies to farmers on complex interactions of various factors involved in small-scale agriculture which can impact levels of *Escherichia coli* in drinking water [45]. In PNG, evidence suggests that a perception of low prevalence of malaria and/or absence of mosquitoes are barriers to high coverage of LLIN use [33, 34]. In education and behavioural change programmes, the improved visualization of novel tools such as BDNs showing how prevalence may vary between populations may improve coverage and uptake of vector control interventions by assisting programmes to target information [34, 46].

The inherent ability of BDN models to represent the reliability of prevalence maps associated with uncertainty in spatial prevalence prediction can be of particular benefit in communicating information to national control programmes [15, 46, 47]. Malaria control programmes need to ensure that disease prevention and control interventions are delivered to areas where prevalence is highest, and being able to visually represent the accuracy of prevalence maps can help guide decisions about efficient and cost-effective targeting of vector control interventions [48]. Spatial prevalence maps such as these can be a useful tool for stratifying a country according to malaria prevalence for designing sub-national control and elimination approaches [4]. Generating spatial prevalence maps using the results of models from which we can represent this uncertainty in predictions, as well as carrying out cross validation on model predictions, make BDN models valuable epidemiological tools for guiding interventions and surveillance [49].

Some limitations are inherent in the work we have presented here. *Plasmodium vivax* may present a particular challenge to malaria control programmes due to the high number of infections in PNG attributed to recrudescence [50] and underestimation of prevalence by surveys which only measure blood-stage infections [14]. The high recrudescence rate complicates the development of ecological models of transmission due to introducing a source of error in estimating covariate effects, and may limit the prediction accuracy of modelling approaches such as those utilized here. In addition to this, using the BDN approach demonstrated here, explanatory variables with continuous data must first be discretized before being used in models, leading to a potential loss of information and subjective decisions regarding the discretization threshold [28]. Random Forest or classification tree modelling approaches which do not require data discretization could be explored as alternatives for future malaria modelling work. Lastly, the approach described in this paper did not incorporate spatial autocorrelation in the models which would make them unsuitable for data in which there is spatial dependency [23]. Future development of BDN modelling methodologies allowing incorporation of data in which spatial dependency exists may be needed in order to accommodate this issue.

## Conclusions

Generating predictive prevalence maps of malaria in large geographic areas in which spatial non-stationarity exists in associations between explanatory variables and the outcome of interest poses challenges to conventional statistical methods such as GLMs. Utilizing novel modelling approaches such as BDNs may help to overcome such challenges and improve the accuracy of spatial predictions for targeted interventions and informing control and elimination programmes. Results obtained from the comparative analysis carried out here, examining the predictive accuracy of BDNs and GLMs found BDNs to perform better in terms of predictive accuracy for malaria in PNG. Future directions may include adapting models to incorporate temporal data to examine spatiotemporal patterns in malaria transmission dynamics, and in incorporating data in which spatial dependency is evident.

## Data Availability

The datasets generated and/or analysed during the current study are not publicly available but are available upon reasonable request.

## References

[1] World Bank. Papua New Guinea overview. http://www.worldbank.org/en/country/png.

[2] Hetzel MW, Pulford J, Maraga S, Barnadas C, Reimer LJ, Tavul L (2014). Evaluation of the global fund-supported national malaria control program in Papua New Guinea, 2009–2014. PNG Med J.

[3] Hetzel MW, Pulford J, Ura Y, Jamea-Maiasa S, Tandrapah A, Tarongka N (2017). Insecticide-treated nets and malaria prevalence, Papua New Guinea, 2008–2014. Bull World Health Organ.

[4] WHO (2015). Global technical strategy for malaria 2016–2030.

[5] Murray CJ, Ortblad KF, Guinovart C, Lim SS, Wolock TM, Roberts DA (2014). Global, regional, and national incidence and mortality for HIV, tuberculosis, and malaria during 1990–2013: a systematic analysis for the Global Burden of Disease Study 2013. Lancet.

[6] Hetzel MW, Saweri OP, Kuadima JJ, Smith I, Ura Y, Tandrapah A (2018). Papua New Guinea malaria indicator survey 2016–2017: malaria prevention, infection and treatment.

[7] Cattani J, Moir J, Gibson F, Ginny M, Paino J, Davidson W (1986). Small-area variations in the epidemiology of malaria in Madang Province. PNG Med J.

[8] Rodríguez-Rodríguez D, Maraga S, Jamea-Maiasa S, Tandrapah A, Makita L, Siba PM (2019). Mapping routine malaria incidence at village level for targeted control in Papua New Guinea. Geospat Health.

[9] Rodriguez-Rodriguez D, Maraga S, Lorry L, Robinson LJ, Siba PM, Mueller I (2019). Repeated mosquito net distributions, improved treatment, and trends in malaria cases in sentinel health facilities in Papua New Guinea. Malar J.

[10] Rodríguez-Rodríguez D, Katusele M, Auwun A, Marem M, Robinson LJ, Laman M (2021). Human behaviour, livelihood, and malaria transmission in two sites of Papua New Guinea. J Infect Dis.

[11] Carter R, Mendis KN, Roberts D (2000). Spatial targeting of interventions against malaria. Bull World Health Organ.

[12] Dalrymple U, Mappin B, Gething PW (2015). Malaria mapping: understanding the global endemicity of falciparum and vivax malaria. BMC Med.

[13] Müller I, Bockarie M, Alpers M, Smith T (2003). The epidemiology of malaria in Papua New Guinea. Trends Parasitol.

[14] Ome-Kaius M, Kattenberg JH, Zaloumis S, Siba M, Kiniboro B, Jally S (2019). Differential impact of malaria control interventions on *P. falciparum* and *P. vivax* infections in young Papua New Guinean children. BMC Med.

[15] Mihretie A, Merkord CL, Bayabil E, Kassa GT, Henebry GM, Lake M (2017). Integrating malaria surveillance with climate data for outbreak detection and forecasting: the EPIDEMIA system. Malar J.

[16] Bhatt S, Cameron E, Flaxman SR, Weiss DJ, Smith DL, Gething PW (2017). Improved prediction accuracy for disease risk mapping using Gaussian process stacked generalization. J R Soc Interface.

[17] Bhatt S, Weiss D, Cameron E, Bisanzio D, Mappin B, Dalrymple U (2015). The effect of malaria control on *Plasmodium falciparum* in Africa between 2000 and 2015. Nature.

[18] Magalhães RJS, Salamat MS, Leonardo L, Gray DJ, Carabin H, Halton K (2014). Geographical distribution of human *Schistosoma japonicum* infection in the Philippines: tools to support disease control and further elimination. Int J Parasitol.

[19] Sturrock HJ, Bennett AF, Midekisa A, Gosling RD, Gething PW, Greenhouse B (2016). Mapping malaria risk in low transmission settings: challenges and opportunities. Trends Parasitol.

[20] Pigott DM, Howes RE, Wiebe A, Battle KE, Golding N, Gething PW (2015). Prioritising infectious disease mapping. PLoS Negl Trop Dis.

[21] Semakula HM, Song G, Achuu SP, Zhang S (2016). A Bayesian belief network modelling of household factors influencing the risk of malaria: a study of parasitaemia in children under five years of age in sub-Saharan Africa. Environ Model Softw.

[22] Onyiri N (2015). Estimating malaria burden in Nigeria: a geostatistical modelling approach. Geospat Health.

[23] Haddawy P, Hasan AI, Kasantikul R, Lawpoolsri S, Sa-angchai P, Kaewkungwal J (2018). Spatiotemporal Bayesian networks for malaria prediction. Artif Intell Med.

[24] Lau CL, Mayfield HJ, Lowry JH, Watson CH, Kama M, Nilles EJ (2017). Unravelling infectious disease eco-epidemiology using Bayesian networks and scenario analysis: a case study of leptospirosis in Fiji. Environ Model Softw.

[25] Korb KB, Nicholson AE (2010). Bayesian artificial intelligence.

[26] Haddawy P, Kasantikul R, Hasan A, Rattanabumrung C, Rungrun P, Suksopee N (2016). Spatiotemporal Bayesian networks for malaria prediction: case study of Northern Thailand. Stud Health Technol Inform.

[27] Chee YE, Wilkinson L, Nicholson AE, Quintana-Ascencio PF, Fauth JE, Hall D (2016). Modelling spatial and temporal changes with GIS and spatial and dynamic Bayesian networks. Environ Model Softw.

[28] Chen SH, Pollino CA (2012). Good practice in Bayesian network modelling. Environ Model Softw.

[29] Landuyt D, Broekx S, D'hondt R, Engelen G, Aertsens J, Goethals PL (2013). A review of Bayesian belief networks in ecosystem service modelling. Environ Model Softw.

[30] Campos-Outcalt D (1989). Health services in Papua New Guinea. Public Health.

[31] Bauze AE, Tran LN, Nguyen K-H, Firth S, Jimenez-Soto E, Dwyer-Lindgren L (2012). Equity and geography: the case of child mortality in Papua New Guinea. PLoS ONE.

[32] Serageldin I, Shluger E, Martin-Brown J (2000). Papua New Guinea-Poverty and access to public services.

[33] Hetzel MW, Choudhury A, Pulford J, Ura Y, Whittaker M, Siba PM (2014). Progress in mosquito net coverage in Papua New Guinea. Malar J.

[34] Pulford J, Oakiva T, Angwin A, Bryant M, Mueller I, Hetzel MW (2012). Indifferent to disease: a qualitative investigation of the reasons why some Papua New Guineans who own mosquito nets choose not to use them. Soc Sci Med.

[35] Hetzel MW, Gideon G, Lote N, Makita L, Siba PM, Mueller I (2012). Ownership and usage of mosquito nets after four years of large-scale free distribution in Papua New Guinea. Malar J.

[36] Hijmans RJ, Cameron SE, Parra JL, Jones PG, Jarvis A (2005). Very high resolution interpolated climate surfaces for global land areas. International Journal of Climatology.

[37] Earthdata Search. 2019. Greenbelt, MD: Earth Science Data and Information System (ESDIS) Project, Earth Science Projects Division (ESPD), Flight Projects Directorate, Goddard Space Flight Center (GSFC) National Aeronautics and Space Administration (NASA). URL: https://search.earthdata.nasa.gov/

[38] Scutari M (2010). Learning Bayesian networks with the bnlearn R package. J Stat Softw.

[39] Wood J, Johnson P, Kirk R, McLoughlin K, Blake N, Matheson F (1982). The genetic demography of the Gainj of Papua New Guinea. I. Local differentiation of blood group, red cell enzyme, and serum protein allele frequencies. Am J Phys Anthropol.

[40] Castelletti A, Soncini-Sessa R (2007). Bayesian networks and participatory modelling in water resource management. Environ Model Softw.

[41] Beresniak A, Bertherat E, Perea W, Soga G, Souley R, Dupont D, Hugonnet S (2012). A Bayesian network approach to the study of historical epidemiological databases: modelling meningitis outbreaks in the Niger. Bull World Health Organ.

[42] Ssempiira J, Nambuusi B, Kissa J, Agaba B, Makumbi F, Kasasa S (2017). Geostatistical modelling of malaria indicator survey data to assess the effects of interventions on the geographical distribution of malaria prevalence in children less than 5 years in Uganda. PLoS ONE.

[43] Ho SH, Speldewinde P, Cook A (2016). A Bayesian belief network for Murray Valley encephalitis virus risk assessment in Western Australia. Int J Health Geogr.

[44] WorldPop (www.worldpop.org - School of Geography and Environmental Science, University of Southampton; Department of Geography and Geosciences, University of Louisville; Departement de Geographie, Universite de Namur) and Center for International Earth Science Information Network (CIESIN), Columbia University (2018). Global High Resolution Population Denominators Project - Funded by The Bill and Melinda Gates Foundation (OPP1134076). The spatial distribution of population density in 2010, Papua New Guinea. https://www.worldpop.org/geodata/summary?id=48152

[45] Hall DC, Le QB (2017). Use of Bayesian networks in predicting contamination of drinking water with *E. coli* in rural Vietnam. Trans R Soc Trop Med Hyg.

[46] Fagerlin A, Valley TS, Scherer AM, Knaus M, Das E, Zikmund-Fisher BJ (2017). Communicating infectious disease prevalence through graphics: results from an international survey. Vaccine.

[47] Spiegelhalter D, Pearson M, Short I (2011). Visualizing uncertainty about the future. Science.

[48] Thawer SG, Chacky F, Runge M, Reaves E, Mandike R, Lazaro S (2020). Sub-national stratification of malaria risk in mainland Tanzania: a simplified assembly of survey and routine data. Malar J.

[49] Tatem AJ, Huang Z, Narib C, Kumar U, Kandula D, Pindolia DK (2014). Integrating rapid risk mapping and mobile phone call record data for strategic malaria elimination planning. Malar J.

[50] Lin E, Kiniboro B, Gray L, Dobbie S, Robinson L, Laumaea A (2010). Differential patterns of infection and disease with *P. falciparum* and *P. vivax* in young Papua New Guinean children. PLoS ONE.

