# Correction to: Spatial prediction of malaria prevalence in Papua New Guinea: a comparison of Bayesian decision network and multivariate regression modelling approaches for improved accuracy in prevalence prediction

**DOI:** 10.1186/s12936-021-03838-4

**Published:** 2021-07-16

**Authors:** Eimear Cleary, Manuel W. Hetzel, Peter M. Siba, Colleen L. Lau, Archie C. A. Clements

**Affiliations:** 1 Research School of Population Health, Australian National University, Canberra, Australia; 2 Swiss Tropical and Public Health Institute, Basel, Switzerland; 3 University of Basel, Basel, Switzerland; 4 Papua New Guinea Institute of Medical Research, Goroka, Papua New Guinea; 5 Present Address: Centre for Health Research and Diagnostics, Divine Word University, Madang, Papua New Guinea; 6 School of Public Health, Faculty of Medicine, University of Queensland, Brisbane, Australia; 7 Faculty of Health Sciences, Curtin University, Bentley, Australia; 8 Telethon Kids Institute, Nedlands, Australia

## Correction to: Malar J (2021) 20:269 10.1186/s12936-021-03804-0

Following publication of the original article [1], the authors noticed that one of the names in the author list was incorrect.

Namely, ‘Peter M. Siba’ had been incorrectly listed as ‘Paul Siba’.

The error has since been corrected in the original article.

The authors apologize for any inconvenience caused.
